# Carriers of Mitochondrial DNA Macrohaplogroup N Lineages Reached Australia around 50,000 Years Ago following a Northern Asian Route

**DOI:** 10.1371/journal.pone.0129839

**Published:** 2015-06-08

**Authors:** Rosa Fregel, Vicente Cabrera, Jose M. Larruga, Khaled K. Abu-Amero, Ana M. González

**Affiliations:** 1 Departamento de Genética, Facultad de Biología, Universidad de La Laguna, La Laguna, Tenerife, Spain; 2 Department of Ophthalmology, College of Medicine, King Saud University, Riyadh, Saudi Arabia; Universitat Pompeu Fabra, SPAIN

## Abstract

**Background:**

The modern human colonization of Eurasia and Australia is mostly explained by a single-out-of-Africa exit following a southern coastal route throughout Arabia and India. However, dispersal across the Levant would better explain the introgression with Neanderthals, and more than one exit would fit better with the different ancient genomic components discovered in indigenous Australians and in ancient Europeans. The existence of an additional Northern route used by modern humans to reach Australia was previously deduced from the phylogeography of mtDNA macrohaplogroup N. Here, we present new mtDNA data and new multidisciplinary information that add more support to this northern route.

**Methods:**

MtDNA hypervariable segments and haplogroup diagnostic coding positions were analyzed in 2,278 Saudi Arabs, from which 1,725 are new samples. Besides, we used 623 published mtDNA genomes belonging to macrohaplogroup N, but not R, to build updated phylogenetic trees to calculate their coalescence ages, and more than 70,000 partial mtDNA sequences were screened to establish their respective geographic ranges.

**Results:**

The Saudi mtDNA profile confirms the absence of autochthonous mtDNA lineages in Arabia with coalescence ages deep enough to support population continuity in the region since the out-of-Africa episode. In contrast to Australia, where N(xR) haplogroups are found in high frequency and with deep coalescence ages, there are not autochthonous N(xR) lineages in India nor N(xR) branches with coalescence ages as deep as those found in Australia. These patterns are at odds with the supposition that Australian colonizers harboring N(xR) lineages used a route involving India as a stage. The most ancient N(xR) lineages in Eurasia are found in China, and inconsistently with the coastal route, N(xR) haplogroups with the southernmost geographical range have all more recent radiations than the Australians.

**Conclusions:**

Apart from a single migration event via a southern route, phylogeny and phylogeography of N(xR) lineages support that people carrying mtDNA N lineages could have reach Australia following a northern route through Asia. Data from other disciplines also support this scenario.

## Introduction

There is wide interdisciplinary agreement on the African origin of Anatomically Modern Humans (AMH) around 200 thousand years ago (kya), and also on the idea that they expanded out of that continent to colonize the rest of the world replacing, with only minor genetic exchanges, the indigenous hominids already present in Eurasia [1,2]. However, there is still inter and intra-disciplinary disagreement about the time and routes used by AMH in their dispersal out of Africa.

Based mainly on the coalescence age of mitochondrial DNA (mtDNA) L3 lineages, most geneticists propose a temporal window of 60–70 kya as the time for the exit, coinciding with the early Last Glacial stage (MIS 4). This hypothesis involves a southern route to Arabia across the Bab al Mandab strait, which, at that time, would have presented a very low sea level [3–6]. Some difficulties with this proposal are: the need of sea strait crossing, the inhospitable climatic conditions in Arabia at that time, the lack of pertinent fossil record along the trail, and the early colonization of Australia. Specially problematic is the date of the arrival of AMH to Australia, the last stage of the initial phase of the AMH colonization of the world, that occurred at least 45 kya [7] attending to the fossil record, but that could be as old as 62 to 75 kya based on genomic aboriginal Australian data [8]. However, all these problems have been overcome by appealing to navigation skills, coastal resource specialization, present-time submerged fossil record, and a very fast spread across coastal India, Myanmar, Malaysia and Indonesia to reach Australasia in time. Recent archaeological studies of Middle Paleolithic stone assemblages in several sites of the Arabian Peninsula [9–11] have added archaeological support to the southern route although entering Arabia during the last interglacial, around 120 kya, much earlier than the dates estimated from mtDNA by the geneticists. It is worth mentioning that a wade ashore across the Bab al Mandeb strait in that period would be more difficult than during a glacial stage.

On the other hand, a northern route by land across the Sinai Peninsula, for the out of Africa migration, is strongly sustained by paleontological and archaeological evidence, as the presence of AMH remains and associated stone material in the Levant around 100 kya [12,13]. The temporal coincidence of this date with an interglacial period would improve the climatic conditions of this corridor facilitating this northern exit. However, in this case, the lack of AMH fossil continuity in the area prompted researchers to consider it as an unproductive exit. Against this idea, recent studies on ancient genomes have detected a basal Eurasian component in the Near East, which diverged prior to the separation of the ancestors of Europeans and Eastern Asians. This finding reinforces the idea that the early presence of modern humans in the Levant was not an unsuccessful episode [14].

At the beginning of this century, studies based on mtDNA complete genomes [15–18] confirmed that only two mtDNA lineages (named M and N), sister branches of the African macro-haplogroup L3 lineages, embraced all the mtDNA variation that exists out of Africa. Based on the phylogeography of M and N in Eurasia, it was proposed that M and N could respectively represent the maternal signals of both a southern and a northern route out of Africa [19]. The huge quantity of data gathered during these years by the paleontology, archaeology and genetics, including genomics and archaeogenomics fields, support that the first modern human colonizers of Australia, carrying mtDNA N(xR) lineages, followed a northern route, across northern Asia and through the Indonesian eastern side of the Wallace line. That reinforces our previous view of the existence of a northern route based on the phylogeny and phylogeography of mtDNA haplogroup N. The goal of this paper is to add further experimental data and putting all this evidence in a coherent picture.

## Material and Methods

### Ethics Statement

Ethical approval was provided by the Ethics Committee for Human Research at the University of La Laguna. Written consent was recorded from all participants prior to taking part in the study.

### Samples

In this study, we collected 1,725 blood/saliva samples from unrelated and healthy Saudi Arabian donors for mtDNA HVR amplification. Only individuals with all their known ancestors born in Saudi Arabia were considered. We also selected 28 samples of western Asian origin (five of them previously published in Maca-Meyer et al. [17]) and 11 of Saudi Arabian origin for mtDNA complete sequencing. Written informed consent was obtained from all individuals.

### MtDNA sequencing

The mtDNA hypervariable regions I and II of 1,725 new Saudi Arabian samples were amplified and sequenced as detailed elsewhere [20]. When necessary, haplogroup diagnostic SNPs were typed using PCR-RFLPs or SNaPshot multiplex reactions [21]. The 1,725 new partial mtDNA sequences have been deposited in GenBank under accession numbers KP960570-KP962294. In addition, complete mtDNA genome sequencing was carried out on 28 western Asian individuals of uncertain or atypical haplogroup adscription. These include the reanalysis of five samples belonging to haplogroup N(xR) previously published in Maca-Meyer et al. [17]. For mtDNA genome sequencing, amplification primers and PCR conditions were as previously published [17]. Successfully amplified products were sequenced for both complementary strands using the DYEnamic ET Dye terminator kit (Amersham Biosciences) and samples run on MegaBACE 1000 (Amersham Biosciences) according to the manufacturer's protocol. The 23 new complete mtDNA sequences have been deposited in GenBank under accession numbers KM245130-KM245152. The five sequences previously published [17] and reanalyzed here have kept their previous GenBank accession numbers (S3 Table).

### Previous published data compilation

Complete and partial sequences belonging to specific haplogroups were obtained from public databases such as NCBI, MITOMAP the1000 Genomes Project and from the literature. We searched for mtDNA lineages directly using diagnostic SNPs, or by submitting short fragments including those diagnostic SNPs to a BLAST search (http://blast.st-va.ncbi.nlm.nih.gov/Blast.cgi). Haplotypes extracted from the literature were transformed into sequences using the HaploSearch program [22]. Sequences were manually aligned and compared to the rCRS [23] with BioEdit Sequence Alignment program [24]. Haplogroup assignment was performed by hand, screening for diagnostic positions or diagnostic motifs at hypervariable regions and at coding regions whenever possible.

We retrieved 623 published complete sequences of Eurasian and Oceanian origin from public databases to build the phylogenetic trees of N(xR) haplogroups (lineages that belong to N but not to its R subclade): N1a3a (n = 29 mitogenomes), X (n = 2), N7 (n = 13), N8 (n = 2), N9 (n = 269), N10 (n = 4), N11 (n = 18), O/N12 (n = 4), N13 (n = 2), N21 (n = 11), N22 (n = 7), A (n = 247) and S (n = 15). To accurately establish the geographic ranges of the relatively rare haplogroups, we searched 73,215 partial sequences (references are in S1 Table) from the literature. A total of 328 of these previously published partial sequences could be unequivocally classified into haplogroups: N7 (n = 13), N8 (n = 33), N10 (n = 71), N11 (n = 58), N21 (n = 113), and N22 (n = 40) (S2 Table). For western Eurasian haplogroups we relied on recent reviews carried out by others: N1 [6,25–29], N2 [6,27–29], N3 [26,28–30], N5 [27,31], and X [6,26,27,32]. In addition, 553 Arabian samples previously published in Abu-Amero et al. [19]) were also included in our study.

### Phylogenetic analysis

Phylogenetic trees were constructed by means of the Network program, v4.6.1.2 using, in sequent order, the Reduced Median algorithm, Median Joining algorithm and Steiner (MP) algorithm [33]. Remaining reticulations were manually resolved. Haplogroup branches were named following the nomenclature proposed by the PhyloTree database [34] (Build 16; http://www.phylotree.org/). Coalescence ages were estimated by using statistics rho [35] and sigma [36], and the calibration rate proposed by Soares et al. [37]. Differences in coalescence ages were calculated by two-tailed t-tests.

### Phylogeographic analysis

In this study, we are dealing with the earliest periods of the out-of-Africa spread, and later demographic growth and expansions most probably eroded those early movements. For that reason, we omitted spatial geographic distributions of haplogroups based on contemporary frequencies or diversities, and used a simple presence/absence of N basal lineages criterion to establish the present-day haplogroup geographic range and the overlapping geographic area of those haplogroups as the most probable center of the old expansion.

### Correlation analysis

To test for correlation between N(xR) haplogroups coalescence ages and their relative geographic distances from Africa we used parametric Pearson tests and modeled a non-parametric Kendall rank-correlation [38] formulating a monotonically decreasing function in which to a geographic increasing longitude value, from Djibouti eastwards to Australia, a decreasing haplogroup coalescent mean age value is associated. The first and last points of this function correspond, respectively, to the empirical coalescent ages of macro-haplogroup L3 and haplogroup S at the Djibouti and Australia geographic longitudes. The upper and lower bounds of this function are marked by the corresponding 95% confidence intervals (95% CI) associated to each mean age point. In the Kendall rank-correlation we consider a concordant pair when, at a given longitude, the 95% CI associated to the model and to the experimental haplogroup coalescent ages overlap, being a discordant pair if they do not. The geographic center of gravity for each haplogroup was estimated as the point at which the segment joining the most distant latitudinal borders and the segment joining the most distant longitudinal borders of the haplogroup geographic range crossed. Maps and geographic coordinates were obtained using Google Earth software (https://earth.google.com).

## Results

### Macrohaplogroup N

Coalescence ages, based on complete mtDNA sequences, for the main branches of macrohaplogroup N(xR), and their present-day geographic distributions are shown in Table 1 and S1–S2 Figs. Haplogroup N11 presents the oldest divergence (around 76 kya) with two main branches, N11a and N11b. N11a is spread in central, western China and Inner Mongolia, and also in southern China and in Makatao from Taiwan [39–42], whereas N11b is found in Philippines [43,44].The second most ancient lineage is N10 (around 66 kya) being mainly detected in southern China, the Tibet and in Lingao from Hainan [39,45]. It is relevant to mention here that, albeit in a smaller proportion, Tibetan and Southeastern Asians, like Filipinos, have introgressed Denisovan-like DNA in their genomes [46,47]. Around 50 kya N(xR) representatives diverged at the same time at very distant geographic areas as western Eurasia (N1 and N2) and Australia (S). Incidentally, as most parsimonious, we propose the Australian N14 lineage [48] as a branch of S1a, sharing 5291 transition with another Australian S sequence (S1 Fig). Later N(xR) spreads, around 40 kya, occurred in a global geographic range from West Asia including North Africa (haplogroup X), southeast Asia (N7 in Cambodia), to northeast Asia (N9) extending also to Australia (O/N12). Other haplogroups as M and R derivatives, also present in Australia, could have reached this continent in that period as a secondary migratory wave. Ancient DNA analyses of an early modern human from Tianyuan cave in northern China, dated around 40 kya [49] and a modern human from western Siberia dated around 45 kya [50], showed that these two individuals already belonged to mtDNA haplogroup R lineages, the main derived branch of macrohaplogroup N. In addition, they carried portions of DNA derived from Neanderthals similar to people present-day in mainland Asia, but lacked of the Denisovan component detected in Negritos of Philippines, Papuans and aboriginal Australians and, at less proportion, in southeastern Asians and Tibetans [47,51], reinforcing the idea that Asian expansions at that period were driven by carriers of derived mtDNA lineages and that the Tianyuan specimen was genetically a fully modern human.

**Table 1:** Age estimates, in thousands of years, for L3, M, and the main branches of haplogroup N.

Haplogroup	This study^1^	Behar et al. 2012		Other authors^2^		n° defining mutations	Geographic range
L3	70.8(52.7–88.1)	67.3 ± 4.4	71.6^h^(57.1–86.6)	78.3^c^(62.4–94.9)	94.3^d^± 9.9	3	Africa
M	48.4(42.0–54.8)	49.6 ± 1.8				3	Asia
N	60.2(46.1–74.2)	58.9 ± 2.4	65.1^b^(52.8–77.8)			5	Eurasia
R	54.5(45.2–65.6)	56.5 ± 2.1	54.5^g^± 2.0			2	Eurasia
N1	51.9(37.1–68.3)	51.6 ± 5.6	54.2^h^(41.3–67.5)			3	West Eurasia, North Africa
N2	48.3(31.5–69.2)	44.5 ± 7.4	50.9^b^(30.5–72.5)			5	South and West Eurasia
N3	11.9(4.0–20.3)		15.4^a^ ±11.9	11.9^f^(4.0–20.3)		17	West Eurasia
N5	35.7(19.8–51.5)	36.7 ± 8.2				7	South and West Eurasia
N7	36.4(22.5–50.9)					7	Cambodia
N8	20.4(9.8–31.6)					12	South China
N9	37.9(37.5–48.7)	45.7 ± 7.9	49.1^h^(34.2–64.6)			1	East Asia
N10	66.4(39.2–93.4)	50.4 ± 6.5	63.4^e^(53.1–74.0)			4	Southeast China
N11	75.9(48.4–104.9)	56.3 ± 3.6				1	Philippines, China, Tibet
O/N12	43.0(26.8–60.1)	52.1 ± 6.4				3	Australia
N13	29.3(16.2–43.0)					13	Australia
N21	17.5(8.7–26.6)	22.4 ± 9.0				7	Indonesia, Malaysia
N22	17.0(8.8–25.5)	25.2 ± 8.8				7	Southeast Asia
A	27.6(19.3–38.3)	24.2 ± 4.9	29.2^h^(19.1–39.8)	33.7^c^(22.4–45.1)		8	Central and Northeast Asia
S	46.8(37.0–56.9)	53.5 ± 5.5				1	Australia
X	31.9(20.7–45.6)	31.7 ± 11.7	33.8^b^(22.5–45.7)			7	West Eurasia, North Africa

1.- Age estimates from complete sequences using rho and the calculator provided by Soares et al. 2009.

2.- a = Derenko et al. 2013; b = Fernandes et al. 2012; c = Fu et al. 2013; d = Gonder et al. 2007; e = Kong et al. 2011; f = Kushniarevich et al. 2013; g = Pierron et al. 2011; h = Soares et al. 2009.

N(xR) haplogroups with the southernmost geographical ranges as N8, N21 and N22 had all significantly more recent radiations than those of Chinese haplogroups N10 (p < 0.0001 in all cases) and N11 (p < 0.0001 in all cases) and the Australian lineages S (p < 0.0001 in all cases) and O (p< 0.0001 for N21 and N22 and p = 0.0074 for N8). These results are inconsistent with a southern route for N(xR). Furthermore, they are also significantly younger (p < 0.0001 in all cases) than the youngest northern Asian haplogroup A (Table 1). It has to be mentioned that, from our analysis of 247 haplogroup A complete sequences (S2 Fig), we have detected 32 new phylogenetic branches of this haplogroup, tentatively represented in red on the A tree. Also inconsistent with the southern route hypothesis is the fact that relative diversities point to an origin in island Southeast Asia for these southern N lineages and recent dispersals westwards into the Malay Peninsula [52].

### The role of the Arabian Peninsula

The southern coastal route hypothesis places the Arabian Peninsula as the initial staging post in the exit of modern humans out of Africa. Our previous mtDNA analyses of Saudi Arabian populations [19,53] evidenced the lack of deep phylogenetic autochthonous Arabian lineages needed to support ancient population continuity in Arabia. On the contrary, the oldest putative indigenous lineages have coalescences ages at the Pleistocene-Holocene boundary and, the present day genetic profile of the Arabian Peninsula fits better as a recipient of relatively recent immigrations than as a source of the pioneer Eurasian colonizers [54]. However, a recent study of N(xR) lineages in the Arabian peninsula has considered the existence of derived N1 branches in the area as relicts of the earliest stage of the southern coastal dispersal of modern humans from the Horn of Africa to the rest of the world [6].

An increase in sample size, including 2,278 Arab mtDNA partial sequences (S4 Table) and the complete mtDNA sequencing of 28 West Asian samples, comprising some rare Saudi and North African lineages (S3 Table and S3 Fig), have not significantly changed our previous results and conclusions [19,53,54]. As before, haplogroups J (21%) and R0a (17%) are the predominant clades. Phylogeographic analysis of these haplogroups [19,53,55] and other lineages with less prevalence in the area as HV1 [56] and R2 [57] seems to indicate population expansions in the Arabian Peninsula mainly after the last glacial maximum coinciding with climate improvement in the area. As for the four R macrohaplogroup complete sequences of Arabian origin analyzed in this study (S3 Fig), one belonged to R0a2c haplogroup, and the other to R1a with an Armenian and an Abkhazian sequence as sister branches [58]. The other two sequences belong to the Indian clades R6 and R8 and were classified as specific R6a1 [59] and R8a3 [60] sublineages. In addition, a rare Georgian sequence has been classified as an R2d lineage (S3 Fig).

Although the bulk of the Arabian sequences (70%) belong to different clades of macrohaplogroup R, 13% percent of Arabian samples belong to haplogroup L, with a clear sub-Saharan African origin. One of the two L Arabian completely sequenced samples was a typical L2a1 lineage with a reversion at the 16309 position. The second is a derived L3i1a sequence, with its closest counterpart observed in Ethiopia (This study and [61]) pointing to a recent importation from northeastern Africa (S3 Fig). Seven per cent of the Arabian samples were assigned to macro-haplogroup M, of which 4% are members of the North African haplogroup M1, and the remaining 3% conform a miscellaneous group of sequences from South, Southeast and Eastern Asian origins and sole representatives of Melanesia (Q1), Madagascar (M32c) or Australia (M42). In particular, the rare Arabian M sample completely sequenced in this study (S3 Fig) belongs to the Indian M42b1 clade, sharing only transversion 95C with a Munda sequence (MUN22) at the same clade. A sister branch of the Indian M42b, with a coalescence time estimation around 55 kya, has spread in Australia [62]. Finally, ten percent of the Saudi Arab sequences were of N(xR) ascription, being the best represented clades the X2 branch of haplogroup X (2.8%) and the derived branches N1a3 (2.1%), N1b (2.0%),N1a1a (1.1%) and I (1.3%) of haplogroup N1.

There is no evidence of deep autochthonous N(xR) clades in the Arabian Peninsula. Moreover, in the few cases in which more than one Arabian sequence is allocated into the same branch their coalescence ages are within the Pleistocene-Holocene period observed for other members of haplogroup R. Furthermore, in the majority of the cases these Arab sequences have sister lineages with deeper roots in the Near East, Iran, the Caucasus or further North. For instance, the two completely sequenced N1a3a Saudi samples (S3 Fig) have a coalescence age of only 11.9 kya. The sole N3a Saudi sample (S3 Fig) can be placed as a derived branch in a tree composed of Iranian and Belarus N3 sequences [28]. Again, the sole X1 Saudi sequence detected (S3 Fig) has a sister counterpart in North Africa, conforming a secondary X1c2 branch with an age of barely 6.5 kya. A close inspection of the complete Arab sequences presented by Fernandes et al. [6] in their N(xR) phylogenetic tree corroborates this scenario. To begin with, there are two sequences from Dubai (DL63 and DL60) that are rooted at 22 kya with a N1e North Asian Buryat, [63]; however the coalescence age for the two Arab members is only 5,216 (103–10,501) ya. In addition, the fact that N1e is a sister branch of haplogroup I, a subclade of N1, deserves mention. The N1a Yemeni isolate JHA114 coalesces at 15 kya with a set of Somalian and Ethiopian sequences. Their N1c (now N1a3a) isolate DL247 from Dubai conforms a clade with European and Caucasian sequences with an age around 18 kya but, our updated N1a3a clade (S3 Fig) conformed by 31 sequences including 6 from the Arabian peninsula, has in fact an age of only around 11.9 (9.2–14.6) kya. A Yemeni sequence (JT196), belonging to haplogroup W, has the oldest coalescence age with sister branches in Turkey and in the Caucasus, around 20 kya. At this point it seems pertinent to mention that a N2a sister branch of the whole W clade has as representative members North Asians Ket [64] and Mansi isolates [65] and an Armenian from the Caucasus [34]. Furthermore, the most ancestral W sequence is that of a Sherpa lineage from the Tibetan highlands [66].

Our detailed analysis of the Arabian N(xR) lineages confirms the lack of ancestral N clades in that Peninsula that could sustain a modern human continuity since the out-of-Africa spread at around 60 kya proposed by geneticists, or around 120 kya according to archaeologists [9–11]. It is of note to mention that a recent study, using complete mtDNA genomes, fully corroborate our negative results also in Yemen [67]. To give a statistical assessment, the N1a3a branch, that joins most Arab lineages with others of western Asian origin, has a coalescence age of only 11.9 ± 2.7 ky being significantly younger (P < 0.0001) than the youngest clade O (43.1 ± 16.5 ky) from Australia. Better than as the cradle of a recent born African modern human, the Arabian Peninsula could be defined as a recent pilgrimage center of worldwide incomers.

### The role of South Asia in the spread of macrohaplogroup N

The main issue of an unique coastal southern route out of Africa was the lack in South Asia of autochthonous N(xR) lineages that are predominant in Australia, the last stage of the out-of-Africa expansion [17,68]. However, the detection of a putative autochthonous N5 lineage in India [31] was enough to reinforce the single southern migration hypothesis, with the important additional assertion that all the three mtDNA founder lineages in Eurasia (M, N, R) travelled together in a unique main expansion. Nevertheless, since then, an impressive amount of mtDNA data on India and West Asia has been published. This new information is in support of a real absence of basal N(xR) autochthonous haplogroups in India. First, no new basic N lineages have been detected. Second, N5 could not be an Indian autochthonous clade based on phylogenetic reconstruction, as it shares transition 1719 with its sister clade N1 that is a haplogroup of undoubted West Asian origin [34], and it has been also found in the Caucasus, Pakistan, Iran and Nepal [26,69–71]. Third, other N lineages detected in India as I, W, X2, or N9a, Y2 and A4, are derived branches of the basal clades N1, N2, X, or N9 and A, of western and eastern Asian origins respectively. Thus, the presence of N lineages in India is better explained as the product of late migration from northwestern and northeastern areas. Even though haplogroup N5 is accepted as an autochthonous Indian lineage, its coalescence age (35.7 ± 8.2) is significantly younger (p < 0.0055) than that of the Australian S lineage (46.8 ± 5.5). This scenario strongly contrasts with the huge presence of autochthonous M [40,72] and R [31,59,60,73] lineages with deep coalescence ages in India. It could be alleged that primary autochthonous N lineages existed in India but became extinct due to genetic drift, but this hypothesis is in contradiction with the fast population growth detected in prehistoric southern Asia [74]. In summary, it seems that the first colonizers of Australia, carrying mtDNA haplogroup N(xR) lineages, could use a route not involving India as a stage. This does not preclude the existence of a southern route across South Asia as proposed by ourselves [17,68] and others [4,75] based also on other mtDNA lineages.

### Early arrival to Australia

The coalescence age of the autochthonous mtDNA haplogroup S, around 50 kya, is compatible with the archaeological dating for human occupation in northern Australia [76], but is out of the MIS 4 glacial period (74–59 kya), when low sea levels would facilitate the travelling from Sunda to Sahul. However, from the genome sequencing of an Aboriginal Australian [8], it was deduced that Aboriginal Australians are descendants of a human dispersal into eastern Asia that occurred as early as 62–75 kya. It was also reported that the mtDNA sequence of that sample belongs to haplogroup O, one of the basic N(xR) lineages in Australia but with later divergence than S. Furthermore, it was confirmed that there was Neanderthal and Denisovan DNA traces in that individual. In particular, the Denisovan component is mainly present in Melanesians, East Indonesians and Negrito from Philippines, compared to other southeastern Asians, and is absent in Andamanese [77]. That is, only populations situated to the east of the biological boundary traced by Alfred Russell Wallace in 1869 seem to consistently share genetic material from Denisovans, pointing to a close relationship among them. At this respect, the presence of mtDNA lineages in Negrito from Philippines, related to the oldest haplogroup N11 deserves special mention. Furthermore, based on genome-wide data, an ancient, Paleolithic, association between Australian New Guinean and Mamanwa from Philippines has been substantiated recently [78].

### Lack of correlation in both routes between haplogroup ages and their geographic distances from Africa

Parametric and non-parametric correlation methods used to test for a negative association between increasing longitude values from eastern Africa to Australia and the coalescence age of present N(xR) haplogroups along the proposed southern and northern routes (Tables 2 and 3), gave non-significant association values in both cases (R = -0.33 and -0.19; Ʈ = 0.02 and 0.15). The most probable causes of this negative results for the southern route are the lack of autochthonous N(xR) lineages in Arabia and South Asia, even accepting N1a3a and N5 as indigenous from the Arabian Peninsula and India respectively, and the young radiations of the southernmost N(xR) haplogroups in southeastern Asia compared to those in Australia. For the northern route, the negative results can be explained by the very old radiation ages of haplogroups N10 and N11 in southern China compared to those of the northern Asian haplogroups A and N9, that, most probably re-expanded during the MIS-3 mid last glacial interstadial (60–25 kya).

**Table 2:** Coordinates for haplogroups assigned to the southern route with observed and expected age values.

Haplogroup	Geographic center	Coordinates	Observed age (Kya)	Expected age (Kya)
L3	Khor Angar (Djibouti)	12°23´N-43°21´E	70.8(52.7–88.1)	70.8(52.7–88.1)
N1a3a	Damqawt (Yemen)	16°34´N-52°51´E	11.9(9.2–14.6)	68.2(56.1–80.0)
N3	Kerman (Iran)	30°00´N-58°00´E	11.9(4.0–20.3)	66.7(54.7–78.7)
N5	Nagpur (India)	21°08´N-79°05´E	35.7(19.8–51.5)	60.9(48.9–72.1)
N7	Phnom Penh (Cambodia)	11°00´N-104°00´E	36.4(22.5–50.9)	54.1(42.9–65.3)
N8	DaNang (Vietnam)	16°00´N-108°00´E	20.4(9.8–31.6)	53.0(42.5–63.5)
N22	Kuching (Malaysia)	01°34´N-110°20´E	17.0(8.8–25.5)	52.4(40.3–64.4)
N21	Samarinda(Indonesia)	01°31´S-118°00´E	17.5(8.7–26.6)	50.2(39.5–60.3)
S	Darwin (Australia)	12°28´S-130°50´E	46.8(37.0–56.9)	46.8(37.0–56.9)

**Table 3:** Coordinates for haplogroups assigned to the northern route with observed and expected age values.

Haplogroup	Geographic center	Coordinates	Observed age (Kya)	Expected age (Kya)
L3	Khor Angar (Djibouti)	12°23´N-43°21´E	70.8(52.7–88.1)	70.8(52.7–88.1)
X	Krasnovodsk (Turkmenistan)	40°10´N-53°00´E	31.9(20.7–45.6)	66.6(57.8–75.7)
N1	Samarkanda (Uzbekistan)	39°37´N-66°58´E	51.9(37.1–68.3)	64.4(54.6–74.1)
N2	Almaty (Kazajistan)	43°13´N-76°51´E	48.3(31.5–69.2)	61.6(71.8–50.8)
A	Urumchi (China)	43°49´N-87°37´E	27.6(19.3–38.3)	58.9(50.0–67.8)
N11	Kunming (China)	24°53´N-102°49´E	75.9(48.4–104.9)	54.5(44.7–64.2)
N10	HoChíMinh (Vietnam)	10°49´N-106°49´E	66.4(39.2–93.4)	53.4(42.3–64.4)
N9	Taiyuan (China)	37°52´N-112°33´E	37.9(27.5–48.7)	51.8(41.2–62.3)
S	Darwin (Australia)	12°28´S-130°50´E	46.8(37.0–56.9)	46.8(37.0–56.9)

## Discussion

Practically all humans out-of-Africa belong to mtDNA macrohaplogroups N or M, both sister branches of L3 African clade. N shows a global Eurasian distribution but most of its lineages everywhere are members of the R subclade. Only in Aboriginal Australians N(xR) lineages reach frequencies over 50% [5,79], and in some regions of East and Central Asia, haplogroups N9 and A can, respectively, exceed 10% [30,39,58,68,80]. In the rest of its geographic range, the presence of N(xR) lineages is residual and represent small younger expansions driven by the later spread of human groups, mainly harboring R derivatives in Western Asia and R and M derivatives in South and East Asia.

**Fig 1:**
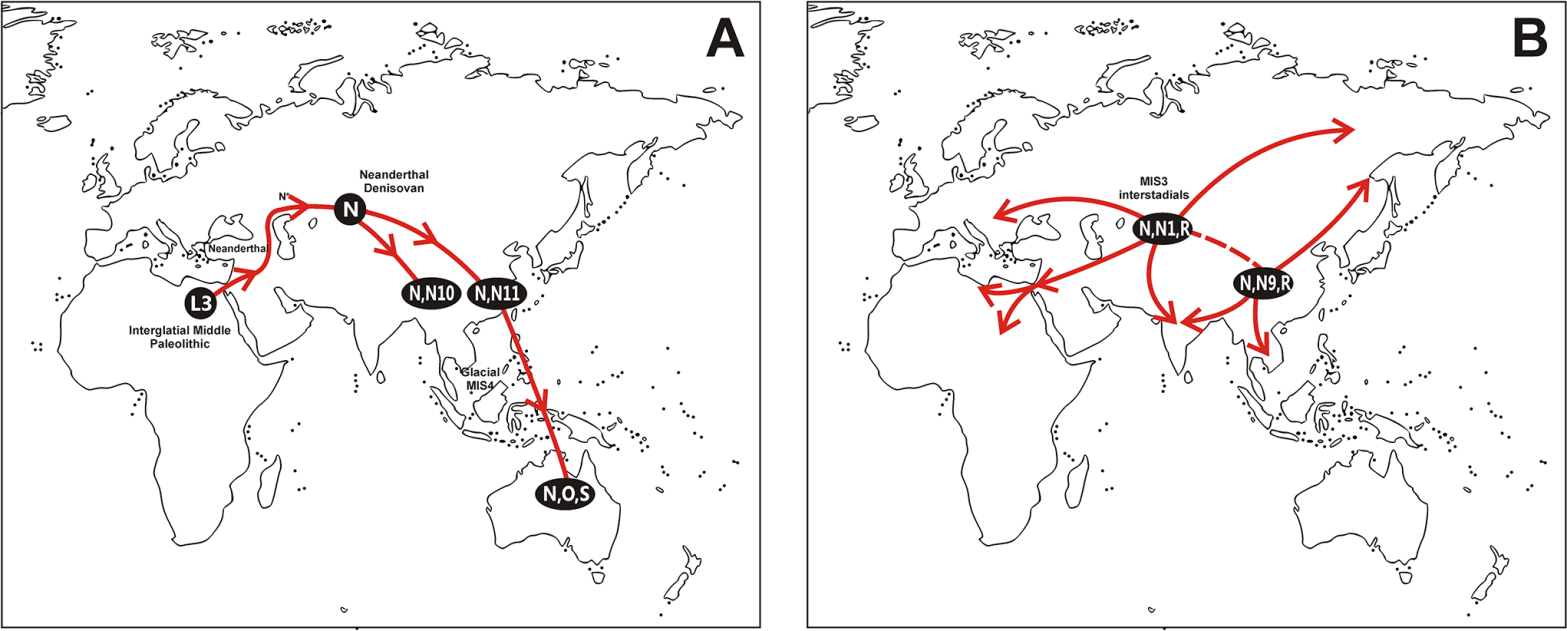
Geographic dispersal routes of (A) AMH out of Africa migration, and (B) secondary worldwide human expansions, deduced from the age and geographic localization of L3 and N(xR) mtDNA haplogroups including Lineages O and S from Australia. Climatic marine isotope stages (MIS) and most probable places of genetic admixture with Neanderthals and Denisovans are depicted. Dotted lines in B mean probable gene flow between populations from different dispersals.

Our phylogenetic and phylogeographic analysis of macrohaplogroup N in Eurasia supports the existence of an additional northern route out of Africa, not involving the Arabian Peninsula or the Indian subcontinent as previously envisaged [17]. This long journey ended in Australia when it was still a part of the Sahul, most probably at the last glacial stage MIS-4 (Fig 1A). On the top of the common L3* trunk, macrohaplogroup N accumulated a stem of five mutations without any known bifurcation. From this fact, it can be deduced that, after the out-of-Africa, the bearers of this lineage seem to have had demographic difficulties and remained as a stagnate population for a long time. So, the first stages of the proposed haplogroup N northern route would be speculative and have to find indirect support on other genetic, archaeological and anthropologic evidences.

### The view from other genetic markers

The first Y-chromosome global studies also confirmed the recent origin of modern humans in Africa and their expansion throughout Eurasia replacing other archaic hominids. It was also deduced that a two routes scenario, one from the Horn of Africa and the other for the Levantine corridor, would be enough to explain the Y-chromosome phylogeography out of Africa [81]. However, subsequent studies, mainly involving the Indian subcontinent, favored the southern route across the Bab el Mandeb strait as the primary migratory passage explaining later northward colonization as its secondary offshoots [75]. In recent years wide genome analyses and whole genome sequencing have been increasingly introduced to further clarify the origin and dispersals of modern humans. The reduced genetic diversity and recombination rates observed in populations situated further and further away from Africa were considered by some authors in support of a recent African origin of modern humans followed by a single gradual colonization of the rest of the world through successive founder steps [82–84]. In addition, it was suggested that the wave of migration out of Africa occurred around 56 kya [85]. Furthermore, another article suggested that the recombination diversity patterns correlate with distance from Africa through a south Arabian, but not a Sinai, route and, within Eurasian populations, recombination distance correlate with distance from Southern India, supporting a single rapid expansion from Africa to eastern Asia with South Asia playing an important role [86]. However, other authors envisaged a more complex scenario, suggesting distinct, early dispersals from Africa [87]. For some authors the earliest dispersal occurred around 130 kya following a southern route to Australia and Melanesia and the later dispersal into northern Eurasia by around 50 kya [88], others situated the out of Africa in a range of 140–80 kya distinguishing subsequent bottlenecks in Europeans and East Asians around 20 kya [89] that could be explained as due to the MIS2 late last glaciation (24–12 kya). The recent explosive human population growth over the last 3,000–4,000 years detected by studies focusing on neutral genomic regions [90,91], is also graphically confirmed at mtDNA level as the most important radiations in the mtDNA phylogenetic tree [34] sprout from secondary nodes with post-Neolithic ages. Perhaps, the patterns of the primary human dispersals, inferred from studies comparing gene diversities in present day populations, should be taken with caution. Anyhow, the northern route deduced from Y-chromosome and global genomic studies is in contradiction, in time and direction with our proposal based on mtDNA haplogroup N(xR). In fact, in a range of 30–50 kya we detect secondary dispersals that colonized western Eurasia and northern Africa (Fig 1B). These movements were named as back-to-Africa migrations by us ([17,92–94]) and others ([32,95–97]). Curiously, returns to Africa have also been detected in Y-chromosome ([98–101]) and genome wide analyses ([102–106]) but with younger ages, ranging from historical times to 23 kya. These discrepancies could be attributed to the real existence of several waves of back-to-Africa migrants, detected preferably by different kinds of genetic markers, to differences in the dating methods or to both causes.

### Out of Africa across the Levant

From a mtDNA perspective, it was the wide radiation of macro-haplogroup L3 in Africa, during a mild climatic period, that prompted the African exit of modern humans to Eurasia [61]. At the beginning, this putative expansion was estimated around 89 ± 69 kya [107], but a later revised mtDNA time scale placed this radiation in an age window between 59 to 95 kya [1,27,49,61], comprising the last phase of a moist interglacial period and the outset of an arid glacial period. In addition, it has to be mentioned that, using a revised genome-wide mutation rate [108], the split between non-African and African populations was situated in a range of 90 to 130 kya. These ranges overlap with the presence of modern humans in the Levant, as attested by the fossil evidence retrieved from the Qafzeh and Skhul caves [109]. It also coincides with a wet climatic period that would facilitate a sub-Saharan Africa northward spread to the Mediterranean shores across the present-day Saharan desert, not only through the Nile Valley but also across Libya and the Maghreb [110–112]. In the same temporal window is the Aterian stone industry that extended overall in North Africa and the Sahara desert, from the Atlantic coast to the Nile Valley and outward into the Levant [113]. Of paramount importance is the evidence that Aterian presents hints of modern human behavior as suggested by the inclusion of ornamental shell beads in their African and Levantine assemblages, and the technological advantage of their stem tools, suitable for hafting [114,115]. Furthermore, affinities between Aterian skulls and Levantine earlier *Homo sapiens* have been reported [116], as well as cranial morphometric affinities between Levantine and later Pleistocene/early Holocene human populations from Australia [117]. All these evidences points to a successful Paleolithic exit through the Sinai Peninsula during the last interglacial period (Fig 1A). Finally, as sea levels would be higher in that time than in glacial episodes, alternative routes involving crossing maritime straits as the Bab al Mandeb through Arabia or the Gibraltar through Iberia would have fewer possibilities of success. This leaves the crossing of the Sinai Peninsula by land as the most plausible gate of exit. During this favorable climatic window, the Mediterranean Africa and the Levantine corridor presented a rather uniform environment that allowed the continuous dispersal into the Near East of small groups of modern humans with close familiar ties. These groups would carry basal L3* mtDNA lineages as their African counterparts. Only two of those lineages have survived till present day, giving the M and N macro-haplogroups that comprise all the non-African extant mitochondrial diversity. The current phylogeography of M and N and their respective coalescent ages of 46 to 53 kya and 54 to 64 kya [27] allow an earliest northward expansion of at least a group carrying a mtDNA lineage that, along the migration route, gave rise to macro-haplogroup N which comprises all the current N lineages, including the derived R characterized by the reversion of the 16223 transition and the presence of the 12705 substitution [17].

### Up to the Caucasus and beyond

There are several possible routes to penetrate to the interior of Asia from the Levant [13], however, archaeogenomics points to the Caucasus as the most probable path (Fig 1A). In effect, the reliable recovery and sequencing of ancient DNA from archaic hominids such as Neanderthal and Denisovan have greatly enlightened their genetic interactions with modern humans. Neanderthal genomic sequencing [118,119], and their comparison with modern humans, detected a limited rate of gene flow (1.5 to 2.1%) from Neanderthals into non-African modern humans before their split into European and Asian groups. As the geographic range of Neanderthals embraced Europe and parts of western Asia, including the Levant, it was proposed that the interbreeding occurred in the Levant where the out of Africa human groups and Neanderthals first met. Further analyses involving more modern human populations demonstrated that Neanderthals contributed significantly more DNA to modern East Asians and Melanesian than to modern Europeans or South Asians [120–122], and that Neanderthals and modern humans could have interacted in a temporal window of 40–90 kya [123]. More recently, it was stated that the introgressed Neanderthal DNA in humans is more closely related to the Mezmaiskaya Neanderthal from the Caucasus than it is to either the Neanderthal from Altai in Siberia or to the Vindija Neanderthals from Croatia [119]. Clearly, this scenario is in conflict with the hypothesis of a single dispersal out of Africa of modern humans through the Bab al Mandeb strait into Arabia, and a sole southern coastal migration through South Asia to southeastern Asia and Australasia. Although the southeastern range of Neanderthals might have extended to the western bank of the Indus River [124], it is difficult to explain why Eastern Asians have more Neanderthal DNA contribution than Europeans and South Asians. On the contrary, these differences in Neanderthal gene flow fit better within the hypothesis of one origin, multiple dispersals and two routes, east and northwards from the Levant. Thanks, again, to ancient DNA studies on hominid remains from Uzbekistan and the Altai mountains, it is unambiguously known that Neanderthals extended their northeastern range to central Asia and South Siberia [125]. Furthermore, using Neanderthal mtDNA sequences in demographic analysis, it was inferred that western and eastern Neanderthal populations diverged approximately 55–70 kya and that there was fragmentation and population turnover in the west, but genetic continuity in the eastern area [126]. Under these circumstances, those modern human groups that went northwards had to coexist with Neanderthals all along the trail. We do not know how the relationship between modern humans and Neanderthals was. Perhaps when the prey was abundant they cooperated and when it was scarce they conflicted. However, it seems sure that modern humans had to pay a sex toll but, in return, they followed the northward tracks through the Caucasus already opened by the Neanderthals. At least, the ancestors of the future Australian colonizers went up to the Altai Mountains in South Siberia where a different hominid, the Denisovans, already coexisted with the Neanderthals (Fig 1A). As with the case of Neanderthals, the genomic [127] and high-coverage genomic sequencing [120] of a Denisovan individual revealed that modern humans and Denisovans also interbred, but this time it mainly affected the ancestors of Australian, Melanesian, East Indonesian and Mamanwa, a Negrito tribe from Philippines [77]. It has been proposed that the geographic range of Denisovans when the introgression could happen was greater, reaching southeastern Asia [127]. However, against that supposition, it is the fact that the analyzed Altaian Denisovans had an extremely low genetic variation at around 70 kya [120], so that if a greater geographic range existed it would be too early to have admixed with modern humans in Southeast Asia. Furthermore, the substantial introgression of Neanderthal DNA into Denisovans [119], and the close relationship of a mtDNA genome of a hominin from Spain, with *H*.*heiderbergensis* morphological resemblance, to the mtDNA of Denisovans [128] might suggest that the Neanderthal and Denisovan geographic ranges had a substantial overlap in the past.

### Down to Australia

Climatic conditions could drive the first N bearers from southern Siberia down to southeastern Asia and from there to Australia (Fig 1A). This most probably occurred during the continental progressive cooling at the MIS4 glacial period (70–55 kya). They could have followed an interior or coastal route as there is evidence for an early presence of modern humans in Central China at least since 80 kya [129], in Southern China around 100 kya [130,131] and in Laos by 50 kya [132]. Similar old dates have been reported for Indonesia [133] and Philippines [134]. The unique mtDNA hints of these movements could be the haplogroup N11 highly divergent branches located in Philippines [43], and in western and central China, including Tibet and Mongolia [39,41], perhaps isolated remains of a greater geographic occupation that was eroded by subsequent human waves. In this respect it is important to call the attention to the fact that all mtDNA radiations that occurred later than 50 kya in other parts of Eurasia were younger than the first colonization of Australia by haplogroup N lineages.

### The secondary radiations

Shortly after this pioneering adventure, there are clear phylogenetic and phylogeographic signs of worldwide secondary mtDNA expansions in which the three Eurasian macrohaplogroups (M, N and R) actively participated (Fig 1B). These later demographic and geographic expansions occurred during the warming interstadial MIS-3 period from 59 to 24 kya. They were particularly impressive in the Indian subcontinent for the M [40,72,105] and R [59,60,135] macrohaplogroups, but also in West Eurasia even affecting eastern and northern Africa as previously envisaged by our studies [17,92–94] and others [32,95–97]. It has to be stressed that in our opinion, the 45 kya expansion from the Levant to Europe proposed by others [136] in fact signals an important northern input affecting both areas (Fig 1B), as it has been suggested previously from the field of archaeology [137–139].

## Conclusions

An unique southern route for the AMH out of Africa migrations has been placed as the most probable path for the journey that drove our species to colonize the entire world. However, as this study demonstrates, an additional Levant northern route is more congruent with available multidisciplinary data. In addition, combined genetic, archaeological and bioclimatic evidence suggest that, although the early anatomically modern human was born in Africa, the nursery of the modern humans that colonized Eurasia, Oceania and the New World might be first at the south Siberia northwest China core and later in Southeast Asia.

## Supporting Information

S1 Fig Basic N haplogroup trees (excepting N1'5, N2, N3, X and A) showing coalescence ages and sample geographic origin. (XLSX)

S2 Fig Tree for haplogroup A based on complete sequences showing coalescence ages and sample geographic origin. (XLSX)

S3 Fig Tree assorting Arabian and western Asian new complete sequences into corresponding haplogroups. (XLSX)

S1 Table References used in the search for basic N haplotypes. (XLSX)

S2 Table Regions, sample sizes and references where haplotypes belonging to haplogroups N7, N8, N10, N11, N21 and N22 were detected. (XLSX)

S3 Table Twenty-three new, and five reanalyzed, complete mtDNA sequences/ of Saudi Arabia or western Asia origin. (XLSX)

S4 Table Haplotypes, coding diagnostic positions analyzed and haplogroup frequencies obtained from a sample of 2,278 Arab individuals. (XLSX)
